# Facilitators and barriers to seasonal malaria chemoprevention (SMC) uptake in Nigeria: a qualitative approach

**DOI:** 10.1186/s12936-023-04547-w

**Published:** 2023-04-11

**Authors:** Nnenna Ogbulafor, Perpetua Uhomoibhi, Emmanuel Shekarau, Jamilu Nikau, Chukwu Okoronkwo, Nadia M. L. Fanou, Ibrahima Marietou Mbaye, Jean-Louis Ndiaye, Andre-Marie Tchouatieu, Abena Poku-Awuku, Corinne Merle, Susana Scott, Paul Milligan, Aminu Ali, Hauwa’u Evelyn Yusuf, Stephen Oguche, Tukur Dahiru

**Affiliations:** 1 National Malaria Elimination Programme (NMEP), Abuja, Nigeria; 2 University of Abomey-Calavi, (UAC), Cotonou, Benin; 3 Université de Thiès, (UoT), Thies, Senegal; 4 Medicines for Malaria Venture (MMV), Geneva, Switzerland; 5 TDR, Geneva, Switzerland; 6 London School of Hygiene & Tropical Medicine, London, UK; 7 Dept of Sociology, Bayero University of Kano, Kano, Nigeria; 8 Department of Sociology, Kaduna State University, Kaduna, Nigeria; 9 Dept of Paediatrics, University of Jos, Jos, Nigeria; 10 Dept of Community Medicine, Ahmadu Bello University, Zaria, Nigeria

## Abstract

**Background:**

SMC was adopted in Nigeria in 2014 and by 2021 was being implemented in 18 states, over four months between June and October by 143000 community drug distributors (CDDs) to a target population of 23million children. Further expansion of SMC is planned, extending to 21 states with four or five monthly cycles. In view of this massive scale-up, the National Malaria Elimination Programme undertook qualitative research in five states shortly after the 2021 campaign to understand community attitudes to SMC so that these perspectives inform future planning of SMC delivery in Nigeria.

**Methods:**

In 20 wards representing urban and rural areas with low and high SMC coverage in five states, focus group discussions were held with caregivers, and in-depth interviews conducted with community leaders and community drug distributors. Interviews were also held with local government area and State malaria focal persons and at national level with the NMEP coordinator, and representatives of partners working on SMC in Nigeria. Interviews were recorded and transcribed, those in local languages translated into English, and transcripts analysed using NVivo software.

**Results:**

In total, 84 focus groups and 106 interviews were completed. Malaria was seen as a major health concern, SMC was widely accepted as a key preventive measure, and community drug distributors (CDDs) were generally trusted. Caregivers preferred SMC delivered door-to-door to the fixed-point approach, because it allowed them to continue daily tasks, and allowed time for the CDD to answer questions. Barriers to SMC uptake included perceived side-effects of SMC drugs, a lack of understanding of the purpose of SMC, mistrust and suspicions that medicines provided free may be unsafe or ineffective, and local shortages of drugs.

**Conclusions:**

Recommendations from this study were shared with all community drug distributors and others involved in SMC campaigns during cascade training in 2022, including the need to strengthen communication about the safety and effectiveness of SMC, recruiting distributors from the local community, greater involvement of state and national level pharmacovigilance coordinators, and stricter adherence to the planned medicine allocations to avoid local shortages. The findings reinforce the importance of retaining door-to-door delivery of SMC.

**Supplementary Information:**

The online version contains supplementary material available at 10.1186/s12936-023-04547-w.

## Background

**Fig. 1:**
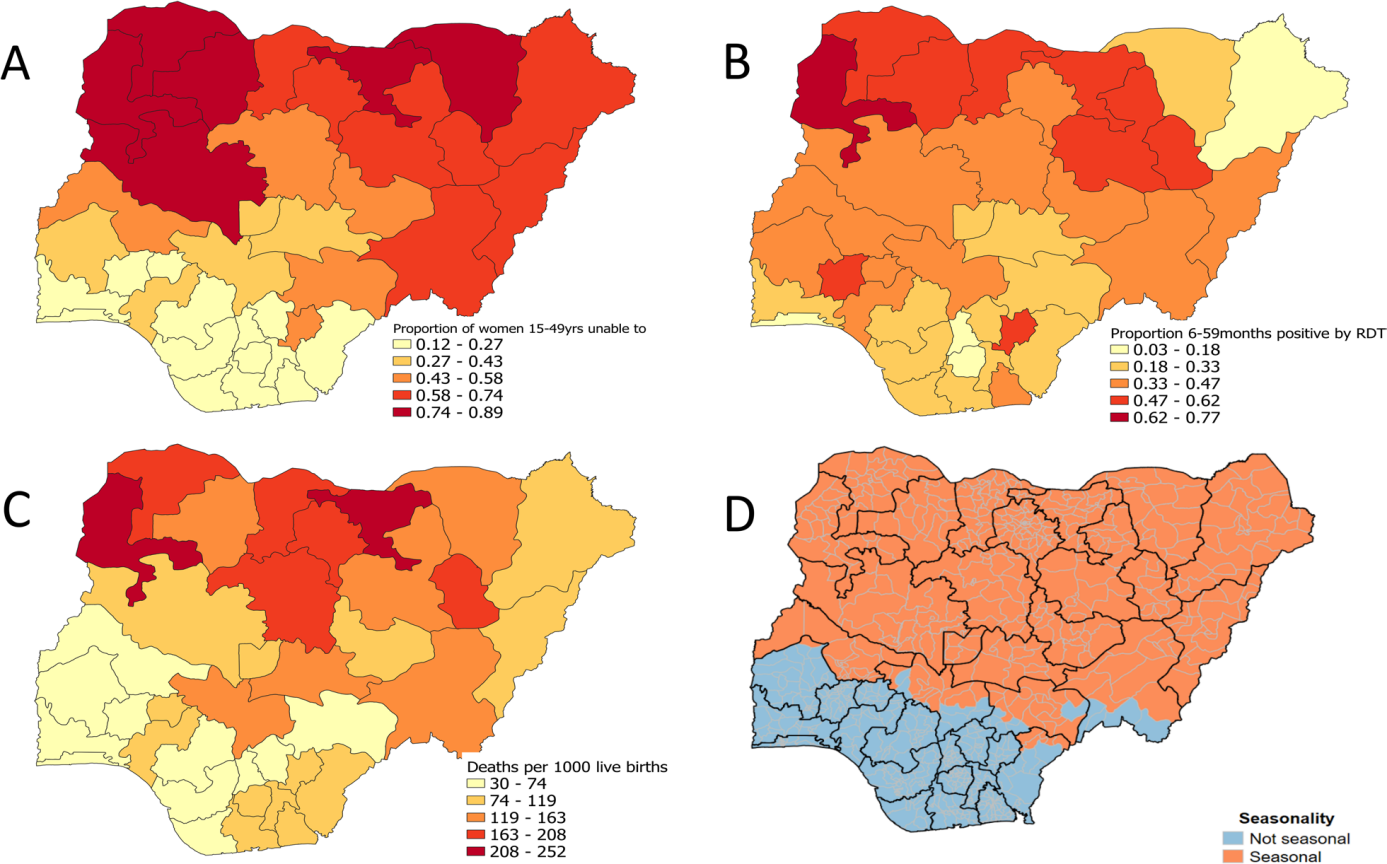
Literacy among women, prevalence of malaria in children, and child mortality, from the 2018 DHS survey [24], and the areas defined as eligible for SMC in the HBHI stratification [21]. **A**: Proportion of women 15–49 yrs able to read; **B** Prevalence of malaria in children 6—59 months (proportion who tested positive by RDT); **C**: Under 5 mortality (deaths per 1000 live births in the 10 years before the survey). **D**: Areas where more than 60% of annual rainfall occurred consistently in 4 consecutive months, considered suitable for SMC in the HBHI stratification

Nigeria accounted for 27% of the world malaria cases and 32% of deaths caused by malaria worldwide in 2020, according to World Health Organization (WHO) estimates [1], most of this burden being in young children. In 2019, Nigeria initiated the High Burden to High Impact (HBHI) approach to malaria control [2], which involves defining optimal combinations of core interventions according to the local epidemiology [3]. The country stratification (Fig. 1) identified 21 of 36 states as suitable for Seasonal Malaria Chemoprevention (SMC).

SMC involves intermittent administration of full treatment courses of anti-malarial drugs during the peak period of malaria transmission each year to children living in areas where malaria transmission is highly seasonal. The drugs recommended for the intervention are sulfadoxine-pyrimethamine plus amodiaquine (SPAQ), administered at 4-week intervals. SMC was shown in clinical trials to prevent 75% of malaria cases during the transmission season [4]. In Nigeria, the current recommendations are for four monthly cycles, but a fifth cycle may be required in some areas, to maintain therapeutic concentration of the drugs throughout the period of the greatest risk of malaria transmission.

**Fig. 2:**
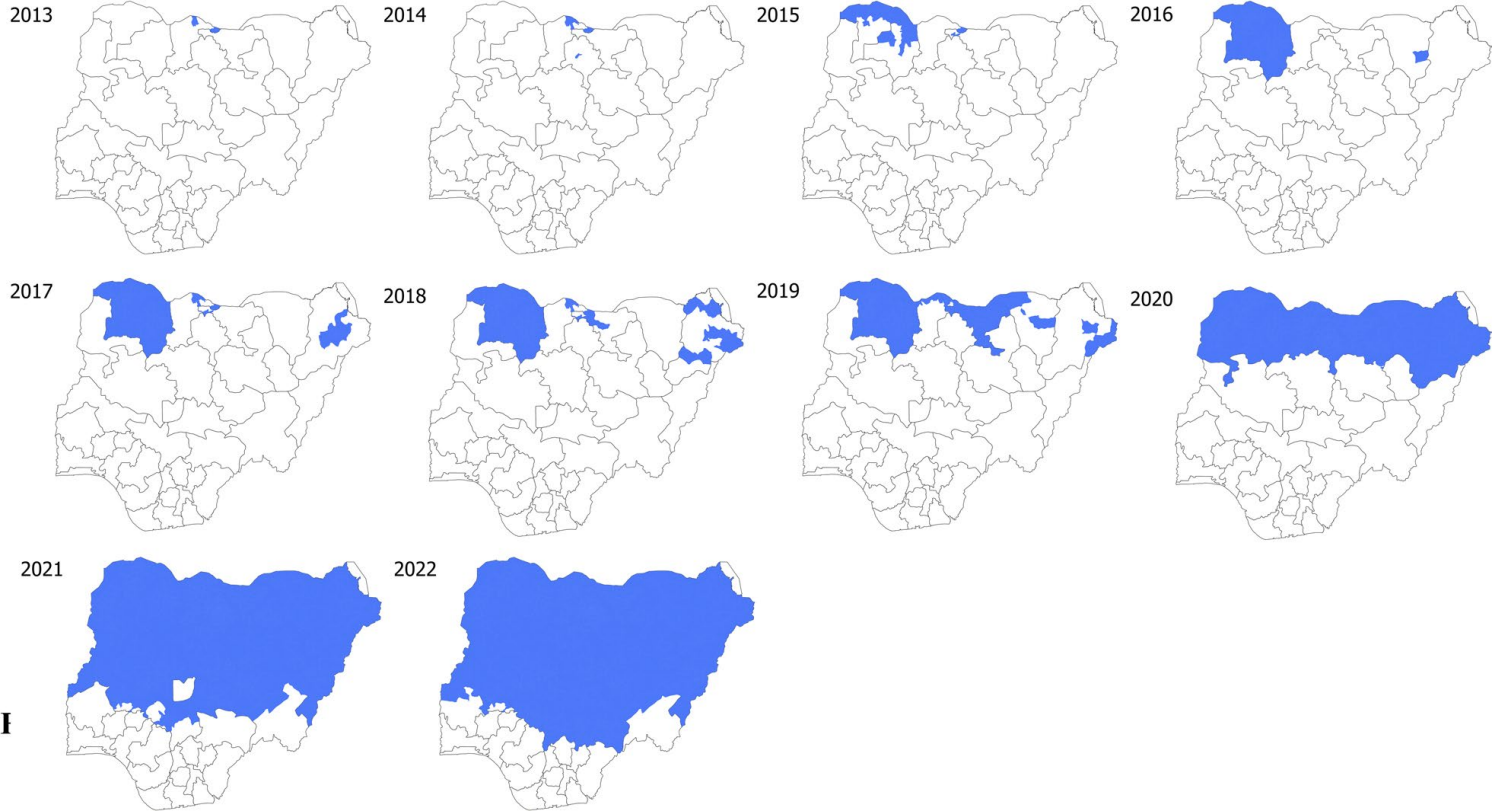
Scale-up of SMC 2013–2022

SMC was adopted by the Federal Ministry of Health as a national malaria control policy in Nigeria in early 2014. After pilot schemes in Katsina state in 2013 and 2014 [5, 6], and Kano in 2014 [7], SMC was introduced in Sokoto and Zamfara states in 2015 and 2016 [8], and its use gradually scaled up, with the intervention implemented in 18 states by 2021 (Fig. 2). In 2021, SMC was delivered over four or five months between June and October by 143,000 community drug distributors (CDDs) to a target population of 23.1million children, with five monthly cycles in Kogi, Nasawara, Plateau states and four cycles elsewhere. In 2022, SMC was planned to be implemented in all 21 eligible states, to a total of 27.1 million children.

Pilot implementation in Katsina (three cycles in 2013 and four cycles in 2014), showed high coverage of SMC could be achieved, and it was reported that SMC was acceptable to communities, 83.9% of eligible children received at SMC at least once, no serious adverse drug reactions were reported, and average economic cost of US$3.98 per child per year in 2013 and $3.77 in 2014 [5]. Evaluation of the scaling up of SMC in seven countries including Nigeria in 2015 and 2016 found that uptake varied but was associated with marked reductions in numbers of malaria cases at health facilities and in the number of malaria deaths in hospital, serious adverse drug reactions were rarely reported, drug resistant parasite genotypes were uncommon but there was evidence of selection for resistance to SP. SMC cost an average of US$3.63 per child per year, and was highly cost-effective [8]. In Nigeria, 76.8% of eligible children received SMC at least once and 54.6% received SMC four times in 2015, and 82.7% and 19.5% in 2016; protective efficacy over 28 days post treatment was 83% in a case–control study in Zamfara state in 2016, and there was an estimated 26% reduction in the incidence of malaria during the transmission season in a sample of health facilities associated with SMC in 2015 and a 25% reduction in 2016. In 2017, SMC was implemented in Sokoto and Zamfara states and parts of Katsina and Jigawa states. Surveys found that 88.6% of children received SMC at least once but only 46.4% received SMC four times, with inequalities in uptake in relation to socio-economic status, especially in Sokoto [9]. Coverage surveys undertaken in 2019, 2020 and 2021, with a modified sampling methodology, showed generally very high levels of coverage in all surveyed areas [10].

Effective delivery of SMC relies on community support and participation. There is increasing recognition of the importance of public engagement in planning all healthcare delivery [11, 12]. SMC programmes have generally been welcomed by communities [6, 13–19], but as SMC programmes are expanded there is a need for robust methods to listen to community perspectives and include them in the design of delivery strategies. At the end of the 2021 campaign, the National Malaria Elimination Programme (NMEP) conducted a qualitative research study to understand community attitudes to SMC, factors facilitating uptake, and barriers, to ensure that community perspectives inform future planning of SMC delivery in Nigeria.

## Methods

### Study setting and selection of study areas

Malaria is endemic in Nigeria occurring throughout the year. The intensity and seasonality of transmission varies considerably across the highly diverse ecological zones. Highly seasonal malaria in Nigeria occurs during and shortly after the period of intense rainfall (three-four months) within the Savanna ecological zones of Derived Savannah, Guinea Savannah, Sudan Savannah and Sahel Savannah. SMC was initially implemented in the Northern states where malaria is most highly seasonal. SMC implementation in Nigeria started as a pilot project in six LGAs (Local Government Areas) of Katsina state in 2013 and 2014. In 2014 the country adopted SMC as a country-wide policy, in those states meeting the eligibility criteria [20]. These areas have the highest burden of malaria and of child mortality in Nigeria [24], Fig. 1. In 2019, more eligible states were added, following stratification through the HBHI initiative in collaboration with the WHO, which defined highly seasonal areas as those where more than 60% of annual rainfall fell in four consecutive months. Thus broadening the original definition (60% of cases in four months or 60% of rainfall in three months) leading to a wider geographical area being eligible for SMC [21]. This led to 21 states (including the Federal Capital Territory, FCT) being considered eligible for SMC (Fig. 1). In 2021, SMC was implemented in 18 of these states, in a total of 389 LGAs, with a targeted population of 23.1million eligible children. Figure 2 shows the gradual scale-up of SMC between 2013 and 2022.

The study was conducted with funding from OPT-SMC project [27], which supports SMC implementing countries to conduct implementation research to improve the intervention’s delivery. NMEP engaged two Principal Investigators (PIs) (from University of Jos and Ahmadu Bello University) to support the study and the OPT-SMC team provided technical input to the study’s protocol. Two experienced researchers in each State conducted the interviews, supervised by the study team.

**Fig. 3:**
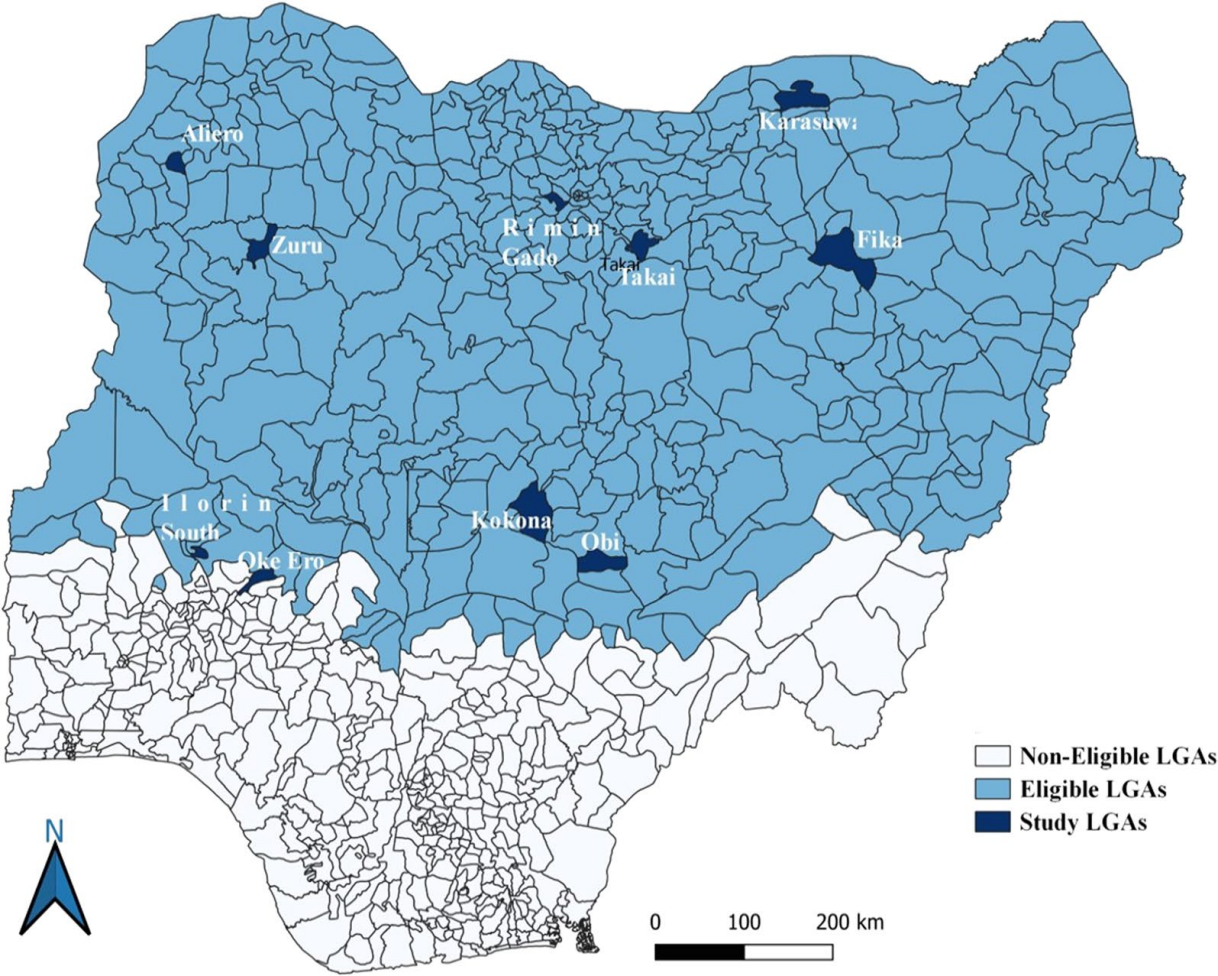
Study locations

Five states were purposively selected, to represent areas supported by each of the three SMC funding partners (Global Fund, US-PMI, and Malaria Consortium philanthropic funding), to include states which started in 2021 and states with more experience of SMC, and to represent the three geopolitical zones out of the six in Nigeria (North West, North East, and North Central). Within each state, LGAs were ranked according to the administrative coverage in 2021 and the LGAs with highest and lowest coverage selected, in order to reflect areas where there were delivery challenges as well as areas where delivery was most successful. In one State, Yobe, the four LGAs with lowest coverage were excluded from the list before selection, due to security concerns. In each selected LGA, rural and urban wards were listed and one ward from each stratum selected with advice from LGA malaria focal persons. Study areas are shown in Fig. 3.

### Protocol development and interview guides

The approach was based on the health belief model [25, 26], aiming to characterize uptake of SMC in terms of perceptions of caregivers and other community members of the risk of the malaria and the severity of its consequences; their views about how well SMC works as an intervention and its potential benefits; what they perceive are the barriers to access, and the ‘cues to action’ or triggering factors that facilitate access to the intervention; and the degree of confidence or self-belief that they will be able to ensure their child receives the intervention (‘self-efficacy’). In-depth Interview (IDI)  guide were developed in consultation with stakeholders and included topics identified in coverage surveys and reflecting the need to explore both demand and supply side factors. The guide for focus group discussions (FDGs) included exploring knowledge of malaria and its consequences, awareness about SMC and the local SMC programme, perceived effects of SMC on children’s health; caregivers’ experience of and attitude towards SMC; barriers to SMC uptake; caregivers preferred mode of delivery/place of administration; and health workers experience and attitude regarding delivery of SMC. Additional File 1, is the In Depth Interview (IDI) guide, it had questions about knowledge of SMC, involvement of the communities, delivery challenges and ways to improve delivery (see IDI guide in Additional file 1).

### Stakeholder engagement

Nigeria operates a three-tier system of government consisting of the Federal, State and Local Governments. At the national level, letters were sent to states, partners and agencies to secure their commitment and cooperation as well as to grant permission to conduct interview with the suitable officer within the state, partners and agencies. At the state level, the State Ministries of Health, State Primary Health Care Boards and Health Departments of the selected LGAs were approached to explain the objectives of the research and to secure their approval and commitment. In each state, meetings were held with the State Malaria programme manager. These engagement meetings were replicated by the research and state teams at the LGA level with the LGA Malaria Focal Persons, who selected an SMC Lead Mother (LM) and a Town Announcer (TA) in each of the chosen wards to help select participants for interviews and focus groups. At community level, influential leaders (religious and traditional) were also consulted.

### Training of interviewers

Interviews were undertaken by two researchers in each State, supervised by two investigators and assisted by five staff of the NMEP. An additional two researchers were responsible for analysis of the interview transcripts. The interviewers were seven staff of the National Population Commission and three university faculty members experienced in qualitative and quantitative field research. Interviewers and analysts were familiar with the local environment and fluent in local languages and English. Interviewer training was held over two days (11–12 November, 2021), facilitated by the principal investigators, staff of NMEP and London School of Hygiene and Tropical Medicine (LSHTM), and one of the data analysts. The training, which included presentations and role play, included a refresher on key features of malaria and malaria control, the implementation of SMC in Nigeria and the steps involved in SMC delivery, the dynamics and process of qualitative interviews, effective facilitation of FGDs, a review of human research ethics in the context of this study, and a detailed review of the interview guides.

### Selection of study participants

In each ward, four Focus Group Discussions (FGDs) were held (one with mothers who could read and write, one with fathers who could read and write, one with mothers who could not read, and one with fathers who could not read, in order to have approximately homogeneous groups [28]). These participants were identified by SMC Lead Mothers (LMs) and town announcers (TAs) chosen by the LGA Malaria Focal Person. The LMs and TAs reside in the communities and well known in their areas. In addition, four in-depth interviews (IDIs) were conducted, with a CDD, a health facility worker, and with two community leaders. This process was repeated in each ward (one rural and one urban ward) in each LGA. In addition, in each LGA, an IDI was held with the malaria focal person, and in each state an IDI was held with the Director of Public Health/Disease Control, and with Malaria Programme Manager. Thus, a total of 16 FGDs and 20 IDIs were completed in each State, a total of 80 FGDs and 100 IDIs. Each FGDs included 8 to 12 participants.

Key informant interviews (KIIs) were conducted with Coordinator of the NMEP, and a representative of each of the partners involved in malaria programme (principal recipient in Nigeria for the Global Fund, WHO, PMI and MC).

### Data collection

All FGDs and interviews were recorded on mobile phones. Five KIIs were conducted via telephone due to COVID-19 restrictions as mandated by their organizations to work from home preventing face-to-face interviews. All FGDs and IDIs were conducted in local languages and later translated into English, while KIIs were conducted in English. The IDIs, KIIs and FGDs were conducted from December 14, 2021 to January 14, 2022. Audio recordings were uploaded to a secure Google drive location along with a verbatim transcript (with names replaced by initials) of each FGD and IDI, and an English translation of each transcript, prepared by each interviewer. For each KII, the recording in English and the transcript was similarly uploaded.

### Data analysis

The data were imported into NVivo 10 for thematic analysis, and the results obtained were presented in narrative statements and subjected to further analysis, using ethnographic summary and content analysis.

### Ethics

The protocol was approved by the National Health Research Ethical Committee (NHREC) at the Federal Ministry of Health, Abuja. Researchers undertook an ethics course provided by TRREE (https://elearning.trree.org/). Administrative approvals were obtained from the State Ministries of Health as well as the Local Government Areas via their Health Departments. In each community, the head of the health facility, assisted by a lead mother and a town announcer, identified potential participants in FGDs and IDIs, and explained the aims and activities of the study in the local language, using an information sheet. A witness was present to attest that the information sheet was explained. Verbal consent was documented through audio recording. Each participant was given two bars of soap and a plastic bucket as incentive, refreshments were provided during the interviews, and for attendance at FGDs transportation costs were provided. Consent was reconfirmed at the start of the FGDs or interview and recorded.

## Results

None of the participants who were approached to join the study refused. In total, 84 FGDs, 100 IDIs and five KIIs were carried out with a total of 1,061 participants. The following themes and sub-themes were identified during analysis: perceived major health problems in the communities; knowledge of Seasonal Malaria Chemoprevention (SMC); extent of SMC uptake and knowledge of malaria and its effect on SMC uptake; perceived effects of SMC on children’s health; caregivers’ experience of, and attitude towards, SMC; barriers to SMC uptake; caregivers preferred mode of delivery/place of administration; and recommendations on how to optimize SMC uptake (Table 1).

**Table 1:** Number of participants in FGDs and KIIs

Location (State)	Focus groups				Key informant interviews	Total participants
	Literate (male)	Literate (female)	Non-Literate (male)	Non-Literate (female)		
Nasarawa	48	48	48	48	20	212
Kano	46	46	48	45	24	209
Kebbi	48	48	48	48	20	212
Kwara	48	48	48	48	20	212
Yobe	46	75	26	45	19	211
National Level					5	5
Total						1061

### Facilitators of SMC uptake

Factors facilitating SMC uptake identified included changes in the drug formulation (the use of sweetened dispersible tablets instead of unsweetened non-dispersible tablets), mode of delivery (which was moved to exclusively door-to-door), recruitment of CDDs from the local area, the involvement of local traditional leaders in the SMC campaign, and CDDs’ ability to build trust and win caregivers’ confidence, were supply-side factors associated with the process of delivery. Demand-side factors included the widespread appreciation of malaria as an important health problem, recognition that SMC is valuable, trust in the local CDDs, and the perceived convenience of the door-to-door approach and the value attached to the opportunity it provides for caregivers to ask questions to the CDD.

### Palatability of the medicine

Sweetened dispersible tablets largely replaced unsweetened non-dispersible formulations by 2017 [8]. Earlier studies found the bitter taste of the unsweetened hard tablets was cited as a reason for caregivers not completing the course of treatment [13]. Some participants commented that the use of sweetened dispersible tablets led to improved uptake of SMC, and may have reduced the risk of children spitting-out the medicine, compared to when unsweetened non-dispersible tablets were used. A key informant said:

*“There has been a lot of improvement in the issue of administering the drugs. The [re]formulation has now made the drugs acceptable to the child and easy for the caregivers to administer because of the improved sweet flavour now. Initially, the parents had to be crushing the drug and adding sugar to it before administering but this has now been taken care of chemically”. (KII/male/Malaria Consortium).*

Children disliked the bitter taste of the previous tablets and tended to spit out some of the medicine when it was administered, reducing the dose received and potentially the efficacy. The dispersible formulation has improved acceptance and effective administration of the SMC.

### Mode of delivery that allows time for explanation and to ask questions

Several participants noted that the use of primarily door to door delivery resulted in improved uptake. When SMC was first introduced in Nigeria, a hybrid approach (combining fixed point and door-to-door delivery methods) was used, this being replaced by exclusively door-to-door delivery from 2016, supported by ‘lead mothers’ who advocate and remind caregivers to administer day two and three doses Caregivers preferred the door-to-door mode of delivery, and its adoption is one of the principal facilitators of effective SMC uptake. All caregivers who spoke about their preferred method of delivery endorsed the door-to-door method. This method is convenient to them in that it does not disrupt domestic tasks. A female caregiver from Nasarawa State stated that a drawback of the fixed-point method was that one might not be able to leave tasks such as washing clothes, to go and line up in the clinic. But with adoption of the door-to-door method, more people now have access to SMC medicine. Similarly, the privacy of the one-on-one interaction between CDDs and caregivers was seen to be important, allowing time for the CDD to explain about SMC and for the caregiver to ask questions. A female caregiver from Yobe State stated that the door-to-door approach:

*“offers greater opportunities for engagement between caregivers and CDDs. The CDDs come to enlighten us on the drugs: how best to use it, the time to administer it and how to notice any development that may arise.”*

Another caregiver from Kano opined that:

*“Door-to-door approach does not affect home chores and the CDDs do either revisit the next day or leave a message with your neighbours if they do not meet you at home. More so, they come early hours in the morning before people go out. This ensures wide coverage and uptake”.*

In Kwara State, a male caregiver argued that the “wide SMC coverage is occasioned by introduction of door-to-door method. The CDDs work well to ensure children get the drugs. They come to us to administer the drug to the kids. So, their drugs reach every household.” further giving reasons why the new mode of delivery facilitates uptake of SMC.

A female caregiver from Yobe State stated that the door-to-door approach:

*“offers greater opportunities for engagement between caregivers and CDDs. The CDDs come to enlighten us on the drugs: how best to use it, the time to administer it and how to notice any development that may arise”.*

CDDs also shared the opinion that change in the mode of delivery has scaled up SMC uptake. One of them from Kwara State stated that “*we go to their place, give them* [SMC medicine] *and make sure they use it in our presence. The following day, lead mothers will go there to confirm if use the drug*.” Another male CDD from Kano “*door-to-door method gives us ample opportunity to sensitize the people to understand the importance of SMC. This has increased uptake*.”

### Endorsement by opinion leaders

Involvement of opinion-leaders (especially religious and traditional leaders) in the SMC campaign was noted as an important factor for promoting acceptance and uptake of SMC. A traditional leader from a rural community in Nasarawa State reported how their involvement in the campaign helped to counter resistance and increase SMC uptake:

*“It is not all the households in his domain that understand the essence of the campaign. While some households are receptive, others do resist, although we usually overcome this by summoning such people to the Palace to further enlighten them on the importance and need for their children to partake in the programme. This works because even before we conclude, they call and give permission to allow the SPAQ to be administered on their children.” (KII/male/traditional ruler).*

Traditional and religious leaders play a critical role in promoting health policies and, as they are trusted and command respect in the community they are often instrumental in mobilising and sensitising the community. Many participants s who spoke during the FGDs commented that the mosque, Islamiyyah (Islamic school) and church are major sources of information/knowledge on SMC medicine. For instance, a male caregiver from Yobe State revealed that:

*“we were told about it [SMC] in the mosque and we informed our wives about the benefits of taking the medicine.”*

Another participant from Kwara State also reported that in each cycle of the campaign, announcement and awareness creation is made in churches and mosques.

### Recruitment of CDDs from the local area

Use of CDDs known and trusted in the community was seen as a key factor influencing SMC uptake. SMC guidelines recommend selecting suitably qualified CDDs from the local area. Many participants s believed that this recommendation was being followed and had been an important factor in promoting increased uptake of SMC. Explaining the selection criteria and how they help to facilitate SMC uptake, a key informant argued that:

*“During the selection of personnel, particularly CDDs, one of the criteria is that the person must be a resident of the community; he/she must have a certain level of education and field experience preferably on SMC campaign or at least other campaigns like polio. The person should be of good/high reputation in the community…. trainings are being done in order to enrich the CDDs with all the vital information needed for the implementation to go well.” (KII/WHO).*

Selection of suitably qualified CDDs from the local area, and the training CDDs undergo, were seen as important in building trust among caregivers and promoting positive attitudes towards SMC. Several caregivers and key informants said that CDDs were selected from their communities and were sensitive to their culture, friendly and diligent in carrying out their duties. In an FGD session with educated mothers in Yobe State, all the participants agreed that CDDs are “*friendly and kind*.” A female caregiver from the same state said that:

*“we trust them because some of them are our children. Explaining the cordial relationship and trust CDDs were able to build, a Focal Person from Kano State reported that “caregivers do give CDDs some gifts to show appreciation. The CDDs come to review meetings with items like eggs, groundnut and some farm produce they get from caregivers.”*

Although several caregivers and key informants said that CDDs were selected from their communities and were sensitive to their culture, friendly and diligent in carrying out their duties, some participants share contrary opinions. In Nasarawa State, for instance, a less educated caregiver argued that “some mothers who do not take the drug for their children feel that the drugs distributors were hostile to them.” A key informant, who works with the Malaria Consortium, also revealed that the guidelines for CDD recruitments are sometimes flouted and pockets of mistrust between caregivers and CDDs were reported. He revealed that there were instances, particularly in state urban cities, where people who were not residents or indigenes of the area are being recruited and those recruited from the urban cities to deliver in neighboring rural communities. Consequently, though rarely, mistrust exist. “*Where we have trust issues is where CDD are not residents/indigenes of the area in question*.”

### Effectiveness of SMC medicines

Although caregivers’ experiences with the SMC varied, many across the study sites had positive attitudes to SMC medicines leading them to accept the treatments. In Nasarawa State, for example, a female caregiver said that:

*“the drug is very good for our children. People were rushing for it. We do not reject it; we come out and accept the drugs.”*

Another caregiver, from a rural area in Kano, stated that:

*“When this drug was brought, we were really happy. It helps a lot. People in this village no longer frequent chemists. Before they started distributing the SMC medicine, we used to go to chemist when our children fell sick to buy paracetamol. If a child took it, the fever might go but it would later come back when the drug finished reacting. But when those drugs (SMC) were brought to us we were really happy. May Allah reward them.” (FGD/Female/Kano State).*

Malaria is the major public health problem in the study sites, implementation of SMC programme was seen as significantly reducing household medical expenses. In Yobe state, knowledge of the medicine and participants’ experiences with it were reported as leading drivers of SMC uptake. A male caregiver from that state said that.

*“people accept the SPAQ. You know we already know about it [effectiveness in preventing malaria]. So, nobody rejects.”*

And a similar point was expressed by a female caregiver in Kano state:

*“We accept the medicine and administer it to our children because we have seen positive changes in them. Malaria has reduced seriously among our children.”*

In Kwara State, another female caregiver revealed that many caregivers

*“who rejected SPAQ in the first cycle of the campaign accepted it in the subsequent one because of the reported efficacy of the medicine in preventing malaria.”*

Not all participants felt SMC is effective in malaria prevention. In Nasarawa State, a less educated participant narrated “a child in their household got infected even after taking the SMC”. She, therefore, questioned the efficacy of the drug.

### Barriers to SMC uptake

Although malaria was widely recognized as an important health problem, and SMC was generally believed to be effective in preventing malaria, concerns were expressed in all five states about side effects of the medicine (whether a general concern that there might be side effects, or experience of problems/symptoms that were attributed to the drugs), and there were concerns about the quality and efficacy of the tablets. Some caregivers were unclear about the purpose of the treatments. Others were put off by the attitude of CDDs which was perceived as unfriendly or impolite. In addition, on the supply side, shortages of SMC drugs were mentioned by both caregivers and CDDs, citing insufficient stocks to treat all eligible children.

### Caregiver mistrust

Despite the high level of acceptance of SMC, there were pockets of resistance in some states, associated with a lack of awareness about the purpose of SMC, and reservations about medicines that are provided free. A caregiver from rural Kano said that

*“those who reject the medicine do so because they think it is harmful to their children.”*

and another caregiver said that

*“some people have a belief that their children could be harmed when they take the medicine. That is why they do not accept it. For instance, some people believe that the medicine can cause infertility in the future.”*

A key informant said despite few problems with rumours, maintaining effective communication was important to counter misinformation:

*“I think we should focus on organizing more of community mobilization activities, because there are no rumors presently on SMC does not mean it cannot happen in the future, the possibility of someone coming up with a strange theory/rumor concerning the programme is very potential, therefore, this is something to watch out to and guard against.”*

Rejection of SMC was also reported in Yobe state by a father, during a male FGD session:

*“there was a time when one man stayed in front of his house and vowed to beat up any person who entered his house to administer the medicine. That man vehemently rejected the medicine.”*

The in-charge of a health facility in Kano state also mentioned instances of refusal reported to them by CDDs:

*“The challenge is that when we send CDDs, some households do not even allow them to enter the house. They sometimes even threaten to beat them up because they say it is family planning tablet; they want to kill people; it’s a coronavirus treatment; etc. This is a challenge.”*

A traditional leader from Nasarawa State reported that:

“There a few challenges. It is not all the households in his domain that understand the essence of the campaign. While some households are receptive, others do resist, although we usually overcome this by summoning such people to the palace to further enlighten them on the importance and need for their children to partake in the programme. This works because even before we conclude, they call and give permission to allow the SPAQ to be administered on their children.”

### Perceived risk of side effects

Concerns about adverse effects of SMC drugs were reported as a barrier to SMC uptake in all the states where the survey was conducted. For instance, when asked why some mothers reject SMC, a caregiver educated to tertiary level from Yobe State said:

“like I said earlier, some side effects manifest in some children. So, some mothers feel reluctant to accept the medicine and administer it to their children.”

The side effects she mentioned in response to an earlier question were body weakness, fever, loss of appetite, and high body temperature. In Kebbi state, a caregiver said:

“some children vomit, have high temperature, and abdominal pain after taking the drug.”

Another caregiver from Kebbi State said that a mother she knows said that her:

“child gets hungry after taking the drug and she does not have food to feed her. Consequently, she stopped administering the medicine.”

And a CDD said that

“the challenge we are facing is just that some mothers say they are not happy with it because of the side effects their children have after they are given the medicine.”

In Nasarawa state, a traditional leader reported that caregivers.

“give their children the medicine, except for those who complain about the side effects: their children get weak when the drug is administered to them.”

### Lack of awareness about the purpose of SMC

Some caregivers said they refused SMC because they were not aware of its purpose, and some CDDs mentioned lack of awareness as a barrier. For example, a caregiver from rural Kano state said during a FGD that the:

“majority of the people who collect the medicine do not use it because they do not know its importance. Some do reject the medicine downrightly when they are offered. They would say if it is something useful, they would not be given.”

And an educated male caregiver from Yobe state said

“people reject the medicine due to lack of knowledge of its efficacy or other personal reasons.”

And in Kebbi state, a caregiver said:

“some time people reject the SMC because they are ignorant about it.”

A father from urban Nasarawa state also mentioned lack of awareness as a major barrier to SMC uptake:

“it is lack of knowledge that deters people from accepting the medicine. Just like COVID-19 [vaccine], people get to realize its efficacy over time and even begin to look for it themselves especially when they see it is working for other people.”

Another father from Kwara state stated that

“some mothers refuse because of their lack of knowledge, they are not informed.”

and a female caregiver from the Yobe state said:

“I think this is related to one’s level of education. If one is well educated, they can give their child this medicine. To me, it all depends on one’s level of education.”

While there was lack of aware of the purpose of SMC among caregivers, overall result indicated that most of them across the study sites are aware. In Kano and Kwara States many participants reported that awareness on the purpose of SMC is high. One of them from the afore-mentioned, for example, said “*majority of us are aware of SMC and we know it is used for kids aged 3 months to 5 years; it is taken within four days and it is effective in preventing malaria*”.

### Local shortages of drugs

Several instances where caregivers were willing to take the SMC but could not, either because the drugs were not readily available or the distributors missed their household, were reported. For example, a key informant from the WHO talked about shortage of SPAQ:

“In fact we even have cases where the drugs could not cover the target population.”

A male caregiver from Nasarawa State also disclosed that

“the drugs are inadequate in this community because it does not go round to all, especially here in Haderi, Kokona LGA.”

This was also reported in Yobe state:

“The reason for some children not getting the drugs is because of shortage. They may start distributing the drugs but before they reach the day four, the drugs have finished and they will not come back until another month. This leads to missing of some doses. Although some people reject the drugs out of will, most people who missed the doses do so because of non-availability of the drugs.” (IDI/Yobe State).

A caregiver from Yobe state said

“some houses were never visited or were missed during the drug distribution.”

Giving reason for shortage of SMC, an LGA malaria Focal Person in Kwara State said that

“there are some areas, such as Oke-Ero, that are hard to reach. Some of these areas are waterlogged, which makes access to these areas for SMC distribution difficult.”

Similarly, a health worker in Yobe State disclosed that the major challenge is getting access to hard-to-reach areas, especially in the rainy season. “*We suffer a lot and the logistics would not be enough for us*.” In addition, disclosing reasons for shortage of SMC medicines, the Kebbi State Director of Public Health revealed that “*there is security compromise in some parts of the state, which affects the distribution of SMC medicines*”.

Shortage of drugs is not a universal problem in the study sites as quite a number of the study participants disclosed that the wide coverage signifies availability of SMC medicines.

## Discussion

SMC was expanded to 21 states in 2022 with a target population of 27.1 million children. To ensure community perspectives inform SMC delivery, the NMEP undertook a qualitative study in five states to understand community attitudes to the intervention. In all study areas malaria was seen as a major health concern, SMC was widely accepted as a key preventive measure, and community drug distributors (CDDs) were generally trusted. Caregivers preferred SMC delivered door-to-door to the fixed-point approach, because in addition to allowing them to continue daily tasks, door-to-door delivery allowed more time for the CDD to explain how to administer the treatments and advise about adverse reactions and to answer questions. However, barriers identified included perceived side effects of SMC drugs, a lack of understanding of the purpose of SMC, and mistrust and suspicions that medicines provided free may be unsafe or ineffective. The use of CDDs from the local area, and endorsement of SMC by local opinion leaders, were reported to be key factors in building trust. Key informants and caregivers reported SMC distributions limited by drug shortages, supplies running out before all children in the community had been treated.

Endorsement by opinion-leaders (especially religious and traditional leaders), and the use of drug distributors from the local community, have been important in influencing acceptance and uptake of SMC. Mistrust and rumours have undermined utilization of healthcare in Northern Nigeria, but other interventions have faced more intense opposition than SMC. Some residents remain adamantly opposed to new drugs and vaccines, violent attacks on vaccinators were among the challenges faced by polio eradication campaign, but resistance to polio campaigns in Nigeria was successfully countered through active and constructive engagement with traditional leaders, imams and Islamic school teachers [22].

Where CDDs were not selected from the local community, trust was difficult to establish and cultural sensitivities may not be respected. A study found that poor attitude of health workers is a serious barrier to accessing healthcare services in Nigeria. Poor attitude withers away service-seekers confidence, which in turn adversely affects acceptance of the services.

CDDs are trained to advise caregivers the correct way to administer SMC medicines, that severe side effects are rare, and what to do if their children experience becomes unwell after treatment. However, there was a widespread perception among caregivers that side effects were a risk this was an important barrier to uptake of SMC. Previous studies on utilization of healthcare services in Nigeria [23] have consistently found that concerns about side effects constitute a barrier to access to any form of treatment, and those who believed they had experienced side effects tend to discontinue or avoid a particular treatment. In Ghana, Antwi et al. [17] also found that perception of side effects was one of the major barriers to SMC uptake.

Palatability of the medicine has improved making treatments easier to administer. Dispersible formulations are recommended by UNICEF and WHO in preference to hard tablets and to liquid formulations. Mixed in a small amount of clean water, they disperse within about three minutes. Dispersible tablets are more expensive to manufacture, and require water-resistant packaging in foil or PVC, but are more palatable and easier to administer than crushed tablets. Sweetened dispersible tablets largely replaced unsweetened non-dispersible formulations by 2017 [8]. Earlier studies found the bitter taste of the unsweetened hard tablets was cited as a reason for caregivers not completing the course of treatment [13].

There was a strong preference for door-to-door delivery. Earlier studies in Senegal noted that a key advantage of door-to-door delivery was that it gave CDDs time to explain the nature and purpose of the intervention to each family which was important to ensure acceptability and adherence [13] and in Niger a switch from fixed point to door-to-door delivery resulted in marked improvement in coverage [8].

The most common reason caregivers gave for not receiving SMC medicines was local shortages of drugs. CDDs also mentioned running out of drugs. While there was no evidence of an overall shortfall in commodities, some hard to reach areas lack the necessary logistics to reach every household. Where local stock-outs did occur, this may have arisen when drug allocations were adjusted during the campaign, providing additional drugs in certain areas based on informal feedback, this can then lead to shortages elsewhere. To address this problem, it is proposed to ensure stricter adherence to annual microplanning allocations. In some areas where there was interruption of SMC delivery due to security problems, this could have been interpreted by caregivers as a shortage of drugs. To maintain delivery in such situations, a strategy that has been used effectively in other areas with security problems, facilitated through WHO with cooperation from the military, is so-called ‘hit and run’ strategies whereby drugs are delivered rapidly at pre-arranged fixed points [29].

Strengths of the study were that it was conducted shortly after the 2021 SMC campaign, in a range of settings across five states, including 1,061 participants interviewed by experienced interviewers. Interviewers and analysts were independent of the NMEP. Limitations were that the study was purely qualitative, it was not possible to quantify how widespread specific issues were; and we did not work in areas with security problems. Analysis of transcripts has not been exhaustive, and a further report is planned.

Actions taken to put these findings into practice included, in 2022, sharing the key points from the study with delivery teams during national and state level training, and then through cascade training to all community drug distributors and others involved in SMC campaigns. To strengthen pharmacovigilance, more involvement of the state-level pharmacovigilance coordinators is needed during implementation to improve completion and submission of individual case safety reports, and at national level, to strengthen collation and investigation of suspected ADRs submitted on paper forms or online. There will be an emphasis on recruiting CDDs from the local community, and the training curriculum will be updated to show SMC teams how to strengthen communication to caregivers on the importance, safety and effectiveness of SMC, during campaigns. To avoid local shortages of SMC drugs, NMEP will ensure stricter adherence to the planned allocations for each facility. The findings of this study reinforce the importance of retaining primarily door-to-door delivery of SMC in Nigeria. Participatory approaches will be need to be incorporated in routine planning to ensure community perspectives continue to inform SMC delivery in Nigeria.

## Supplementary Information

**Additional file 1.** In Depth Interview Guide. This file contains the questions and prompts used by interviewers during data collection. The guide was developed based on a literature review and pilot testing.

## Data Availability

The transcripts for all the interviews are available from National Malaria Elimination Programme, Abuja, Nigeria. Interested persons can contact the corresponding author.

## Abbreviations

SMC: Seasonal malaria chemoprevention
NMEP: National malaria elimination programme
CDD: Community drug distributor
FGD: Focus group discussion
IDI: In-depth interview
KII: Key informant interview
WHO: World Health Organisation
HBHI: High burden to high impact: a targeted malaria response
LGA: Local Government Area
SP-AQ: Sulfadoxine-pyrimethamine plus amodiaquine
NHREC: National health research ethical committee
TREE: Training and resources in research ethics evaluation
UNICEF: United Nations Children’s Fund
PMI: President’s malaria initiative
NAFDAC: National agency for food and drug administration and control in Nigeria

## References

[1] WHO (2021). World Malaria Report 2021.

[2] NMEP. National Malaria Strategic Plan 2021–2025. Federal Ministry of Health, Abuja, Nigeria, 2020.

[3] WHO (2019). High burden to high impact: a targeted malaria response.

[4] Meremikwu MM, Donegan S, Sinclair D, Esu E, Oringanje C (2012). Intermittent preventive treatment for malaria in children living in areas with seasonal transmission. Cochrane Database Syst Rev.

[5] Oresanya O. An implementation trial to explore the feasibility, effectiveness, acceptability and cost of a community based system for seasonal malaria prophylaxis (SMC) in selected LGAs in Katsina State, Northern Nigeria, 1st Malaria World Congress; 2018 Jul 1–5; Melbourne, Australia. https://www.researchgate.net/publication/327977197

[6] Strachan CE, Kana M, Martin S, Dada J, Wandera N, Marasciulo M (2016). The use of formative research to inform the design of a seasonal malaria chemoprevention intervention in northern Nigeria. Malar J.

[7] Ward A, Guillot A, Nepomnyashchiy LE, Graves C, Maloney K, Omoniwa OF (2019). Seasonal malaria chemoprevention packages with malnutrition prevention in northern Nigeria: a pragmatic trial (SMAMP study) with nested case-control. PLoS ONE.

[8] ACCESS-SMC Partnership. Effectiveness of seasonal malaria chemoprevention at scale in West and Central Africa: an observational study. The Lancet. December 2020;396(10265):1830–40. 10.1016/S0140-6736(20)32227-3. 10.1016/S0140-6736(20)32227-3 PMC7718580 33278936

[9] GiveWell. Seasonal Malaria Chemoprevention in Nigeria: coverage surveys 2017, [internet]. 2018 Available https://files.givewell.org/files/DWDA%202009/Malaria%20Consortium/SMC_in_Nigeria_Coverage_surveys_2017.pdf

[10] Federal Ministry of Health. National Malaria Elimination Programme (NMEP) LQAS Survey Report, 2019-2021. FMOH, Abuja, Nigeria, 2021.

[11] Brunton G, Thomas J, O’Mara-Eves A, Jamal F, Oliver S, Kavanagh J (2017). Narratives of community engagement: a systematic review-derived conceptual framework for public health interventions. BMC Public Health.

[12] Ankomah SE, Fusheini A, Derrett S (2021). Barriers and facilitators of patient-public engagement for health system improvement in sub-Saharan Africa: a systematic scoping review. Health Policy Open.

[13] Ba EH, Pitt C, Dial Y, Faye SL, Cairns M, Faye E (2018). Implementation, coverage and equity of large-scale door-to-door delivery of seasonal malaria chemoprevention (SMC) to children under 10 in Senegal. Sci Rep.

[14] Diawara F, Steinhardt LC, Mahamar A, Traore T, Kone DT, Diawara H (2017). Measuring the impact of seasonal malaria chemoprevention as part of routine malaria control in Kita. Mali Malar J.

[15] Barry A, Issiaka D, Traore T, Mahamar A, Diarra B, Sagara I (2018). Optimal mode for delivery of seasonal malaria chemoprevention in Ouelessebougou, Mali: A cluster randomized trial. PLoS ONE.

[16] Tine RC, Ndiaye P, Ndour CT, Faye B, Ndiaye JL, Sylla K (2013). Acceptability by community health workers in Senegal of combining community case management of malaria and seasonal malaria chemoprevention. Malar J.

[17] Antwi GD, Bates LA, King R, Mahama PR, Tagbor H, Cairns M (2016). (2016) Facilitators and barriers to uptake of an extended seasonal malaria chemoprevention programme in Ghana: a qualitative study of caregivers and community health workers. PLoS ONE.

[18] Ansah NA, Malm K, Chatio ST, Ansah P, Tampuulo S, Awuni D (2016). (2016) Evaluation of the impact of implementation of seasonal malaria chemoprevention on morbidity and mortality in young children: a qualitative study in Northern Ghana. Am J Trop Med Hyg.

[19] Shehu UL (2017). Malaria preventive practices and acceptability of seasonal malaria chemoprevention among caregivers of under five children in rural and urban communities of Kano, Nigeria, 2017. Am J Trop Med Hyg.

[20] WHO (2012). Policy Recommendation: Seasonal Malaria Chemoprevention (SMC) for *Plasmodium falciparum* malaria control in highly seasonal transmission areas of the Sahel sub-region in Africa.

[21] WHO (2020). Stratification and analysis for optimizing mix of interventions and resource prioritization.

[22] Nasir S-G, Aliyu G, Ya’u I, Gadanya M, Mohammad M (2014). From intense rejection to advocacy: how muslim clerics were engaged in a polio eradication initiative in Northern Nigeria. PLoS Med.

[23] Ali A. Islamic jurisprudence, agency and contraceptive use among Hausa women in Kano State, Nigeria. PhD Thesis; Department of Sociology, University of Ibadan; 2021.

[24] National Population Commission (NPC) [Nigeria] and ICF (2019) Nigeria Demographic and Health Survey 2018. Abuja, Nigeria, and Rockville, Maryland, USA: NPC and ICF.

[25] Rosenstock IM (1974). Historical Origins of the Health Belief Model. Health Educ Monogr.

[26] Janz NK, Becker MH (1984). The health belief model: a decade later. Health Educ Q.

[27] SMC Alliance. OPT-SMC: Optimizing SMC by Building Capacity for Delivery and Evaluation [Internet]. 2020 [cited 2023 Apr 4]. Available from https://www.smc-alliance.org/opt-smc-optimizing-smc-by-building-capacity-for-delivery-and-evaluation

[28] Roller MR, Lavrakas PJ (2015). Applied qualitative research design: a total quality framework approach.

[29] Nkwogu L, Shuaib F, Braka F, Mkanda P, Banda R, Korir C (2018). Impact of engaging security personnel on access and polio immunization outcomes in security-inaccessible areas in Borno state, Nigeria. BMC Public Health.

