# Supplementary material for: Facilitators and barriers to seasonal malaria chemoprevention (SMC) uptake in Nigeria: a qualitative approach
Source: Malar J. 2023 Apr 11;22:120. doi: 10.1186/s12936-023-04547-w (PMC10088202; doi:10.1186/s12936-023-04547-w)
Supplement: Supplementary file 1 — Additional file 1. In Depth Interview Guide. This file contains the questions and prompts used by interviewers during data collection. The guide was developed based on a literature review and pilot testing. [file 12936_2023_4547_MOESM1_ESM.docx]

**Facilitators and Barriers to Uptake of Seasonal Malaria Chemoprevention Programme in Nigeria: A mixed method approach**

**ADDITIONAL FILE 1**

**IDI Guide**

**IDIs with Community Drug Distributors (CDDs)/Health Facility Workers, Community and Religious Leaders, SMC staff at LGA, State and National level**

**Informed consent**

Dear participant,

This study is being carried out by ____________________ of ____________________________________________________. I am conducting a study on **“Facilitators and Barriers to Uptake of Seasonal Malaria Chemoprevention Programme in Nigeria: A mixed method approach”**. The purpose is to understand, in your own way how and why some mothers accept the treatments for their children while others refused. The findings from this research would be useful in providing information to the government and other stakeholders on how to improve the delivery of SMC programme/services. After completion of this study, the findings from the research will be used to inform policy makers at national, state, local government and facility level on the current standard of care and where improvement in quality SMC programme.

Your participation is totally voluntary and your responses will be treated with utmost confidentiality. You are not expected to disclose your name or personal identity. Withdrawal from the study at any time is allowed. Your sincere and honest answers would be highly appreciated. Thank you.

Please sign below if you are willing to participate in this study.

_____________________________ _______________________________

Signature of respondent Name of interviewer

**Introduction**

Good day, you are welcome to this discussion. I am _________________________________________________________

From___________________________________________________________________________________________________________I am carrying out research on **“Facilitators and Barriers to Uptake of Seasonal Malaria Chemoprevention Programme in Nigeria: A mixed method approach”**. This research is being carried out to get information about your expectations, experiences and attitudes of SMC services provided to you. After completion of this study, the findings from the research will be used to inform policy makers at national, state, local government and facility level to improve the overall delivery and quality of SMC services. I would like you to introduce yourself and freely express your experience during your participation with SMC campaigns.

Thank you for participating.

**Questions:**

1. What do you know about SMC? **Ask** for: the target age group (i.e. eligible age group), preventing children from getting malaria, curing malaria, helps eliminate malaria from the community, number of months required for treatment, timing of treatment during the 4 months of rainy season). Who are the direct beneficiaries of SMC?
2. What is the target coverage of each SMC campaign by ward/LGA? How often should each campaign take place? How long should each campaign last?
3. Who are the different people involved in the implementation of SMC campaign? Explain the roles of these people.
4. Please, can you explain the planning of the activities of the last organized SMC campaign? Is the programme being implemented exactly the same as designed? **Probe:** how are CDDs being recruited, selected, trained, assigned to various LGAs/wards. Did you notice any favoritism in the selection of CDDs? Was there any effective supervision from the LGA or State or Federal officials? Was there any communication line between CDDs and State officials when the CDDs face problems in the field?
5. Is the community receptive of the programme? Are the fathers, mothers/caregivers happy with the programme? Why? Why not?
6. How satisfied are you with the remuneration? Did you face any challenges in the field during the campaign? Can you describe/explain those challenges? How were you able to overcome them?
7. What can you say about SMC program in this ward/LGA/State? What can you say about how SMC is being implemented in this ward/LGA/State?
8. Do mothers/fathers usually accept this drug for their children? How do you know that? Do some/most/all mothers accept the drug and kept without giving to their children? Do mothers complete the whole treatment course even in your absence? How do you ensure they completed the entire dosage? Do you think the mothers/fathers trusted you? Any monitoring mechanism?
9. What can you say about the attitudes of mothers/caregivers regarding SMC programme? Do mothers/caregivers show any form of support to the programme? If yes, in which ways are they showing support? Probe: welcoming you to their homes? Friendly with you? Ask reasonable questions about SMC or the drug treatments?
10. Do caregivers perceive SMC to be good for their children or effective for prevention of malaria? What is your opinion for the programme? Should it still continue to give SPAQ to children or they should stop? Should they take it to other wards/LGA/States or stop it in this ward/LGA/State?
11. Based on your experiences, what do you think the government should do to improve the SMC campaign? What would be your recommendations?
12. What do you consider to be the best things about SMC? What should be encouraged? How? By whom?
13. Do you have any questions for us?
